# Aberrant type 2 dopamine and mu-opioid receptor availability in autism spectrum disorder

**DOI:** 10.1007/s00259-025-07620-5

**Published:** 2025-10-18

**Authors:** Tuomo Noppari, Jouni Tuisku, Lasse Lukkarinen, Pekka Tani, Nina Lindberg, Emma Saure, Hannu Lauerma, Jari Tiihonen, Jussi Hirvonen, Semi Helin, Johan Rajander, Juha Salmi, Lauri Nummenmaa

**Affiliations:** 1 Turku PET Centre, University of Turku, Turku, Finland; 2 Turku University Hospital, Turku, Finland; 3 Department of Psychiatry, Helsinki University Hospital, Helsinki, Finland; 4 Department of Forensic Psychiatry, Helsinki University Hospital, Helsinki, Finland; 5 Clinicum, Department of Public Health, University of Helsinki, Helsinki, Finland; 6 Psychiatric Hospital for Prisoners, Health Care Services for Prisoners, Turku, Finland; 7 Department of Clinical Neuroscience, Karolinska Institute and Center for Psychiatry Research, Stockholm, Sweden; 8 Department of Forensic Psychiatry, University of Eastern Finland, Niuvanniemi Hospital, Kuopio, Finland; 9 Neuroscience Center, University of Helsinki, Helsinki, Finland; 10 Department of Radiology, Turku University Hospital, Turku, Finland; 11 Radiopharmaceutical Chemistry Laboratory, Turku PET Centre, University of Turku, Turku, Finland; 12 Acceletor Laboratory, Turku PET Centre, Åbo Akademi University, Turku, Finland; 13 Unit of Psychology, Faculty of Education and Psychology, University of Oulu, Oulu, Finland; 14 Department of Psychology, University of Turku, Turku, Finland

**Keywords:** Autism, Dopamine, Opioid, Positron emission tomography, MOR, D2R

## Abstract

**Purpose:**

Opioid and dopamine receptor systems are implicated in the pathoetiology of autism, but in vivo human brain imaging evidence for their role remains elusive.

**Methods:**

Here, we investigated regional type 2 dopamine and mu-opioid receptor (D2R and MOR, respectively) availabilities and regional interactions between the two neuromodulatory systems associated with autism spectrum disorder (ASD). In vivo positron emission tomography (PET) with radioligands [11 C]raclopride (D2R) and [11 C]carfentanil (MOR) was carried out in 16 adult males with high functioning ASD and 24 age and sex matched controls. A whole brain voxel-wise analysis was tested with Student´s t-test and regional group differences in D2R and MOR receptor availabilities as total and separate were tested with linear mixed models also examining the associations between regional receptor availabilities with correlations.

**Results:**

There were no group differences in whole-brain voxel-wise analysis of D2R, but ROI analysis revealed a lower overall mean availability in striatum of the ASD compared to controls. Post hoc regional analysis revealed reduced D2R availability in nucleus accumbens and globus pallidus of the ASD group. The whole-brain voxel-wise analysis of MOR revealed cuneal/precuneal up-regulation in the ASD group, but there was no overall group difference in the ROI analysis for MOR. MOR down-regulation was observed in the hippocampi of the ASD group in a post hoc analysis. Regional correlations between D2R and MOR availabilities were weaker in the ASD group versus control group in the amygdala and nucleus accumbens.

**Conclusions:**

These alterations may translate to disrupted modulation of social motivation and reward in ASD.

**Supplementary information:**

The online version contains supplementary material available at 10.1007/s00259-025-07620-5.

## Introduction

Autism spectrum disorder (ASD) is a developmental neuropsychiatric disorder with disabling difficulties in social communication and interaction and restricted and repetitive behavior [1]. ASD is strongly heritable with a complex genetic background [2, 3], but its neurochemical basis remains poorly understood. Aberrancies in different neuroreceptor systems [4] have been proposed to underlie ASD, but human studies have yielded only limited evidence for the role of specific neuroreceptor systems. Animal studies and neuropharmacological evidence, however, suggest that the endogenous dopamine and opioid systems might be the key molecular pathways underlying ASD [4–10].

Dopamine is released from the ventral tegmental area (VTA), which projects to the prefrontal cortex (PFC) and nucleus accumbens (NAcc) of the ventral striatum, forming the mesocorticolimbic circuit (MCL) that controls reward and motivated behavior [5, 11]. Dopamine released from substantia nigra pars compacta (SNpc), in turn, projects to the dorsal striatum (DS), forming the nigrostriatal (NS) pathway, controlling goal-directed and habitual behavior [5]. Dopamine receptors are divided into two main groups D1- (D1R, D5R) and D2-receptors (D2R, D3R, D4R), of which D1R and D2R moderate locomotion, reward, reinforcement, learning, and memory [12]. D1R are expressed as postsynaptic receptors widely across the brain and especially in the striatum [6, 13], while D2R is additionally expressed also as presynaptic auto-receptors, which modulate dopamine release in the synapses [14]. The use of dopaminergic agents, such as cocaine and methamphetamine, is associated with smaller social networks and impaired emotion recognition and emotional empathy [15]. Cocaine users also show difficulties in the theory of mind [16, 17] and display diminished emotional engagement and reward in social interaction [18, 19]. Functional magnetic resonance imaging (fMRI) studies in humans have suggested that the dysfunction of the MCL is associated with ASD [20–23]. Human positron emission tomography (PET) studies have further shown deviant dopamine signaling in ASD compared to controls, but the results have been controversial [24, 25]. There is also support for dopamine dysregulation in ASD from genetic studies [26]. Finally, dopamine antagonist medications such as risperidone and aripiprazole may alleviate autism symptoms, including irritability and stereotypic behavior [4–6, 27].

The endogenous opioid system controls a wide variety of human functions, from basic homeostasis and antinociception to emotions, social attachment, and reward [9, 28–31]. Neuropeptide beta-endorphin is released from hypothalamic nuclei and mesolimbic structures, projecting to other hypothalamic and corticolimbic structures, frontal lobes, brainstem, and periaqueductal grey [9, 28]. Beta-endorphin binds to three different opioid receptors, mu- (MOR), delta- (DOR), and kappa- (KOR), which are differentially distributed across the cortical and subcortical areas. Opioid receptors are widely expressed in the brain as postsynaptic receptors, but also as modulatory presynaptic auto-receptors [32]. Since the late seventies, opioid system dysfunctions have been proposed to be linked with autism [7, 9, 33, 34], and more recently, MOR knock-out mice were shown to manifest autism-related behaviors [10]. In animals, mu-opioid agonists also influence the social approach and withdrawal behavior depending on the dose, duration of dosing, and the social context [8]. Clinical studies in humans have shown that opiate addicts in maintenance treatment display deficits in social inference, emotion recognition and theory of mind [35–37]. Moreover, opioid antagonists may improve hyperactivity and restlessness symptoms in autism [4, 38]. The endogenous opioid system aberrancies in ASD have not been validated with any human imaging studies.

The interaction of the dopamine and opioid systems is critical for reward processing [39–42]. D2R and D1R are both expressed in the striatum and current theories suggest their opposing role in reward and aversion learning [14, 43]. Opioid mediated reward in turn depends on MOR activation in VTA, NAcc, amygdala, hippocampus, globus pallidus (GP) and medial frontal cortex (MFC) [44]. In animal microdialysis studies, mu-opioid agonists in the VTA increased the basal level of dopamine in the striatum, whereas antagonists decreased it [45, 46]. In human [11 C]carfentanil PET studies, the dopaminergic drug amphetamine has raised mu-opioid levels in the striatum, frontal cortex, thalamus, insula, and anterior cingulum (ACC) and cerebellum [47, 48]. Multiligand PET studies have confirmed that D2R and MOR availabilities correlate positively with each other in the striatum [49, 50],, and that this linkage may be disrupted in specific conditions such as morbid obesity [50]. Dysfunction of this opioid and dopamine modulated reward-system is associated with ASD and relates to aberrant social reward and motivation [8, 51, 52].

Here, we used in vivo PET with radioligands [11 C]raclopride (binding to D2R) and [11 C]carfentanil (binding to MORs) to compare regional receptor availabilities and the interactions between the receptor systems in ASD individuals and controls. We found that ASD is associated with striatal D2R basal availability downregulation and disruption of D2R and MOR interaction.

## Materials and methods

### Participants

Sixteen individuals with high-functioning ASD (mean age = 30 years, SD = 5, range = 23–42 years) and 24 controls (mean age = 29 years, SD = 9, range = 20–49 years) participated in the study. All participants gave informed, written consent and were compensated for their participation. The ethics board of the Hospital District of Southwest Finland approved the protocol, and the study was conducted in accordance with the Declaration of Helsinki. The study also obtained a research permit from the Hospital District of Helsinki and Uusimaa since the ASD group participants were largely recruited from this area. For minimizing confounding factors and maximizing statistical power, the study was run using a male-only sample with a restricted age range (20–50 years). General exclusion criteria for both groups were current use of narcotics, current abusive use of alcohol (over 14 units per week), severe axis I psychiatric illnesses, autoimmune illnesses, current medical conditions, and the standard MRI contraindications. Structural brain abnormalities that are clinically relevant or could bias the analyses were excluded by a consultant neuroradiologist.

Inclusion criterion for the ASD group was a valid autism spectrum disorder diagnosis (DSM-5 299.00). Participants in the ASD group were volunteers from the Helsinki and Turku University Hospital Neuropsychiatric Clinics, one participant was also recruited from Neuropsychiatric Clinic Proneuron, in Espoo. Diagnoses were verified by a neuropsychologist (JS), neurologist (TN), and psychiatrist (PT) following DSM-5 criteria based on patient history, all accessible information from birth records, well-baby clinics, and school healthcare. Autism Diagnostic Observation Schedule-Second Edition (ADOS-2) assessment [53] was also performed by trained, research-reliable clinical psychologist (ES) to quantify the autism severity in this group. The ASD participants did not have any opioid or dopaminergic medication. Clinical information and characteristics of the ASD participants is found in Table S1.

Inclusion criteria for the control group were no history of neurological or psychiatric disorders. The control participants were screened for medical conditions from their patient histories and their use of prescribed medication was double-checked from the Finnish medical database. The control participants did not have any opioid or dopaminergic medication. Clinical information and characteristics of the control participants is found in Table S1.

All participants completed the Autism Spectrum Quotient (AQ) questionnaire for the evaluation of ASD-related traits (ASD group mean = 28, range = 23–42, SD = 6 and Controls group mean = 12, range = 4–27, SD = 5) [54, 55]. None of the participants had previous or current severe mental disorders based on the SCID-I interview [56]. The education of all the participants was classified into three different categories: (1) primary school, (2) secondary degree, (3) university degree. All the participants were also screened for their clinical status, with comprehensive general blood laboratory exams, including a urinary test for the use of narcotics.

The sample size was calculated based on comparable experiments on brain neurotransmission differences between groups [57]. In these experiments statistically significant differences in neuroreceptor binding could be established between two groups of 13 and 14 individuals with an effect size of d = 1.30. A priori power calculations with the G*Power software suggest that the predicted effects will be observed with a power of 0.90 with sample sizes exceeding 14.

### Experimental design

PET imaging was carried out with General Electric (GE) Healthcare Discovery VCT (ASD *n* = 2, Controls *n* = 17) and Discovery 690 PET/CT (ASD *n* = 14, Controls *n* = 7) scanners in Turku PET center. Spatial resolution is similar (~ 5 mm FWHM) for both scanners [58, 59]. We measured MOR availability with high-affinity agonist radioligand [11 C]carfentanil [60] and D2R availability with agonist radioligand [11 C]raclopride, with the highest affinity to D2R, but also high affinity to D3R and low affinity to D4R [61, 62]. Radiotracer synthesis has been described previously [57, 63]. Both radioligands were administered as a rapid bolus injection, after which the radioactivity in the brain was measured for 51 min. Injected doses were 245 ± 11 MBq for [11 C]carfentanil and 254 ± 11 MBq for [11 C]raclopride). The [11 C]carfentanil and [11 C]raclopride PET imaging were performed on the same day >2.5 h apart. All PET images were reconstructed using 13 timeframes (3 × 1 min, 4 × 3 min, 6 × 6 min; total of 51 min). High-resolution anatomical T1-weighted images (TR 9.8 ms, TE 4.6 ms, flip angle 7º, 250 mm FOV, 256 × 256 voxel matrix with 1 mm^3^ isotropic voxel size) were obtained with 3 T PET-MRI scanner (Philips Ingenuity TF PET-MR device, Philips Healthcare) for reference and normalization purposes. The PET-MR was not available for the lengthy dual-ligand scans for logistic reasons as it was used for clinical scans and other research projects necessitating MR at that moment.

### PET preprocessing and modelling

PET images were preprocessed in MATLAB (The MathWorks, Inc., Natick, Massachusetts, United States) using Magia pipeline [64] (https://github.com/tkkarjal/magia), which utilizes SPM12 (The Wellcome Trust Centre for Neuroimaging, Institute of Neurology, University College London, https://www.fil.ion.ucl.ac.uk/spm/software/spm12) in the PET data motion correction and image registration and FreeSurfer (version 6.3, https://freesurfer.net) in an automated region of interest (ROI) delineation. We used altogether twelve MOR binding ROIs involved in reward, motivation and socioemotional processing extracted from MRI by using FreeSurfer (striatal: caudate, globus pallidus (GP), NAcc, and putamen, extrastriatal: amygdala, thalamus, dorsal anterior cingulate cortices (dACC), rostral anterior cingulate cortices (rACC), OFC, posterior cingulate cortex (PCC), insula and hippocampus) [8, 23, 30–32, 44, 49, 51, 65–68]. Since raclopride binds reliably only in the striatum [69], we preferred the striatal ROIs from these to measure D2R binding [70–73], only with the exception of including thalamus and amygdala because of their striatal connections and relevance in ASD [23, 51, 52, 70, 74–79]. Reference tissues volumes (cerebellum for [11 C]raclopride and occipital cortex for [11 C]carfentanil) were automatically adjusted to account for specific radioligand binding, as described previously [64].

Specific regional binding of [11 C]raclopride and [11 C]carfentanil was quantified as binding potential relative to non-displaceable binding (BP_ND_) using the simplified reference tissue model (SRTM) [80]. Parametric BP_ND_ images were also calculated for voxel-level analysis with basis function implementation of SRTM (bfSRTM) with 300 basis functions. Lower and upper bounds for theta parameter were set to 0.082 1/min and 0.6 1/min for [11 C]raclopride and 0.06 1/min and 0.6 1/min for [^11^ C]carfentanil. Before the parametric image calculation, the dynamic PET images were smoothed using a Gaussian kernel with 4 mm full width at half maximum to reduce the effect of noise in voxel-level bfSRTM fit. The resulting parametric images were further normalized into MNI152 space and smoothed again using a Gaussian 4 mm filter.

### Statistical analysis

Full-volume analysis of the normalized and smoothed [11 C]raclopride and [11 C]carfentanil images was carried out with SPM12 using a two-sample Student’s t-test, while controlling for scanner type. [11 C]carfentanil analysis included the whole brain volume, whereas voxel-wise [11 C]raclopride analysis was restricted to striatum, as BP_ND_ for this ligand can be reliably estimated only within this subregion [69]. The results were corrected for multiple comparisons by using false discovery rate (FDR) at *p* < 0.05.

Statistical analysis for the ROI data was performed using R software (version 3.5.1, The R foundation, Vienna, Austria) and linear mixed-effects models (LMMs), by including a random intercept to control for the potential differences between PET scanners. Before the LMM analysis, all BP_ND_ values were log-transformed to ensure normality. Normality of the model residuals was evaluated with the Shapiro-Wilk test and checked visually with density and Q-Q plots. Homogeneity of variance was verified separately for both scanners with Levene´s test. The results were corrected for multiple comparisons using Holm’s method. The results are presented as percentage difference in BP_ND_ and regression effect estimates with 95% confidence intervals and p-values. We first tested for overall significant differences between the groups by including group as a fixed factor and participant, ROI and scanner type as random effects, allowing their intercepts to vary freely:

$$\\\begin{array}{c}BPnd\sim1\:+\:group+\\(1\vert participant)+(1\vert scanner)+(1\vert roi)\\\end{array}$$

As our primary interest with D2R availability was the striatum, the striatal ROIs were analyzed first separately and then together with the thalamus and amygdala ROIs [69].

We then set up post-hoc analysis for assessing group differences in each ROI separately, by specifying group as a fixed factor and scanner type as a random effect in LMM:

$$BPnd\sim group\:+(1\vert scanner)$$

The association between AQ scores and regional radioligand availability were analyzed with LMM, with AQ and age as a fixed factor and scanner type as a random effect:

$$BPnd\sim AQ\:+\:age+(1\vert scanner)$$

Associations between regional [11 C]carfentanil and [11 C]raclopride BP_ND_ were estimated using Pearson correlations. Group differences in the regional associations in D2R/MOR availability were tested using Fisher’s z-test. A standard neuroimaging tool for matrix correlations, Mantel’s test [81–83] was used to examine the similarity of the regional correlation structures of D2R/MOR availability between the groups.

### Meta-analysis

To summarize the current data on striatal dopamine PET imaging studies in ASD we conducted a meta-analysis. A PubMed search was conducted on Oct. 3rd, 2023, using the keywords “autism spectrum disorder”, “PET”, “positron emission tomography” and “dopamine”. Moreover, a complementary search with Google Scholar was conducted to confirm that no studies were missing. Finally, we screened three of the latest review articles on the issue to ensure the full coverage of our research [24, 84, 85]. Altogether, we found nine potentially eligible articles [72, 74, 86–92], but only six included requisite data on striatal ROIs. Two of these six articles used the same data, so only one was included. Together with our results presented here a total number of six articles were included in the meta-analysis. All the articles were evaluated by three independent researchers of our group (TN, LN, JT) for inclusion in the meta-analysis. There was only one previous study using [11 C]raclopride tracer [72] measuring D2R availability, whereas the other studies used [18 F]FDOPA [87, 89–91] measuring presynaptic dopamine synthesis capacity or D1R binding [11 C]SCH23390 [88] as tracers (see Table S2 for more details). As one of the articles presented required data only in their supplemental figure and it was also not readily accessible from the authors, we used plotdigitizer tool to extract baseline values from the accessible figure [72]. Based on reported striatal group means, standard deviations and sample sizes we calculated the standardized mean difference (SMD, Cohen´s d) for each study and a pooled sample size weighted SMD with R software metafor package.

## Results

**Fig. 1:**
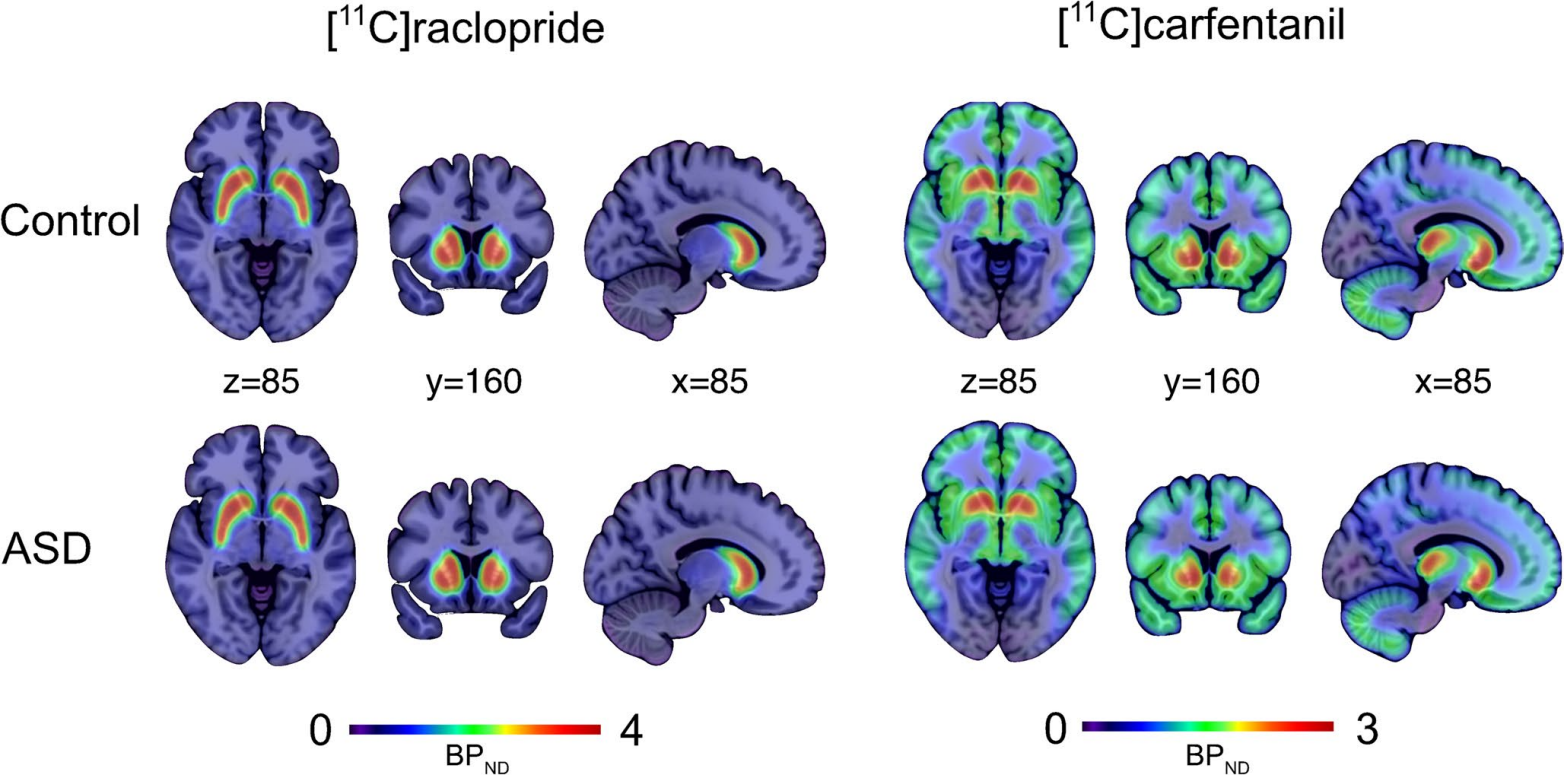
Mean [11 C]raclopride and [11 C]carfentanil BP_ND_ in the control and ASD groups

**Fig. 2:**
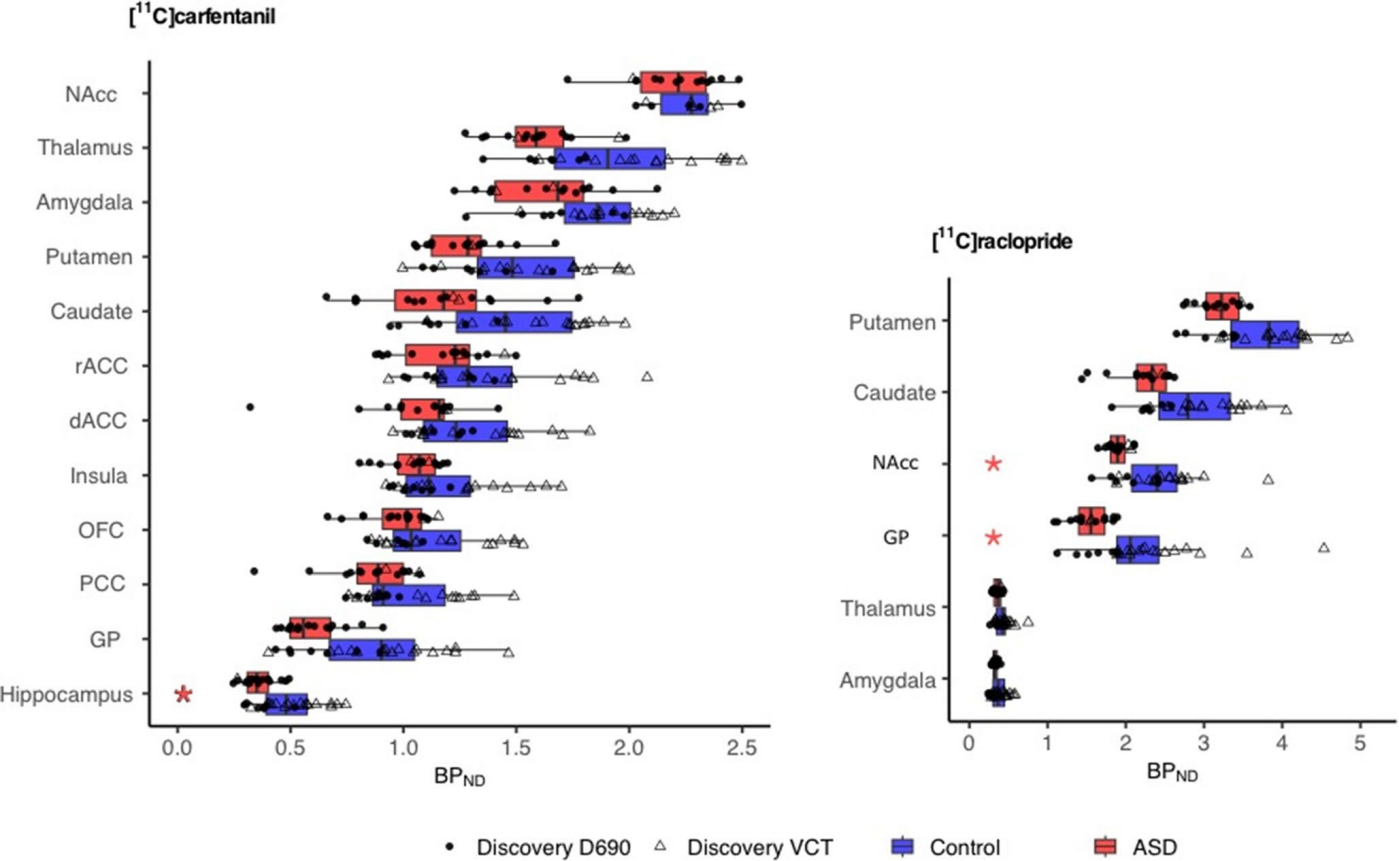
Group-wise binding potentials of [11 C]raclopride and [11 C]carfentanil BP_ND_ in the predefined ROIs. The blue and red boxes mark the 2nd and 3rd quartiles, with the crossline marking the median of the data. Upper and lower whiskers mark 1.5 interquartile ranges from the box. Data with the two different scanners is marked with circles (Discovery 690) and triangles (Discovery VCT). Significant post-hoc differences between the groups are marked with an asterisk

Mean group-wise [^11^C]raclopride and [^11^C] carfentanil binding maps are shown in Fig. 1. Group-wise regional binding potentials for the radioligands are shown in Fig. 2.

### D2R system

**Fig. 3:**
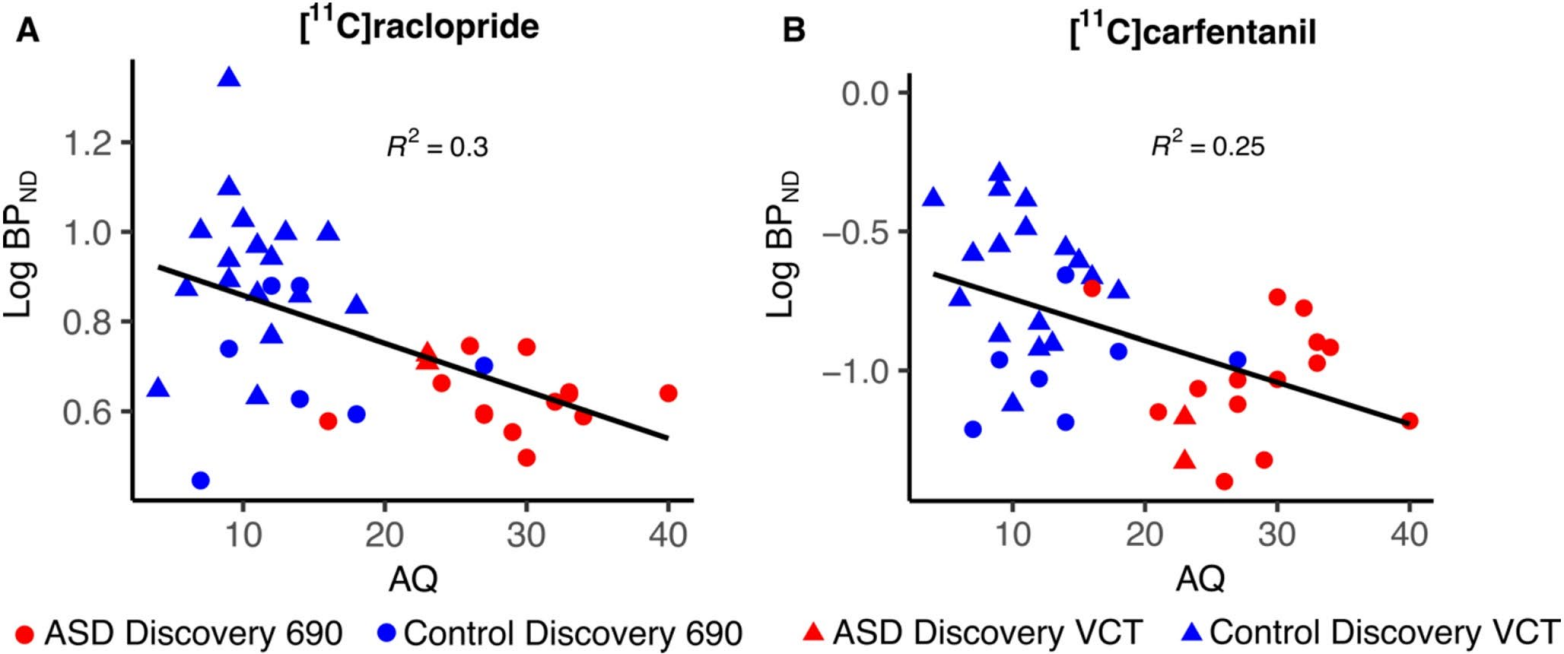
Association and regression line between AQ scores and [11 C]raclopride BP_ND_ in the NAcc across the ASD and control groups (**A**). Association and regression line between AQ scores and [11 C]carfentanil BP_ND_ in the hippocampus (**B**). Data with the two different scanners is marked with circles (Discovery 690) and triangles (Discovery VCT)

Whole-brain voxel-wise analysis did not reveal any significant group differences with [11 C]raclopride. The analysis with striatal ROIs revealed significant overall downregulation in D2R availability of the ASD group (Estimate 0.097, 95% CI [0.004,0.190], *p* = 0.048). A complementary analysis, including extra-striatal regions thalamus and amygdala, revealed no group differences. Separate regional post-hoc analyses showed 13% lower BP_ND_ in the NAcc (Estimate 0.118, 100x(e^0.118^ – 1) = 13%, *p* = 0.034, 95% CI [0.013,0.222], Shapiro *p* = 0.062) and 19% lower BP_ND_ in globus pallidus (Estimate 0.175, 100x(e^0.175^ – 1) = 19%, *p* = 0.048, 95% CI [0.008,0.343], Shapiro *p* = 0.230) (Fig. 2, Table S3). BP_ND_ in the NAcc was also negatively correlated with AQ scores across all participants with one AQ point increase predicting a 0.5% decrease in NAcc BP_ND_ (Estimate − 0.005, *p* = 0.058, Shapiro *p* = 0.253, 100x(e^−0.005^ – 1) = 0.5%), but this effect was not significant when the groups were analyzed separately (Fig. 3). The regional post-hoc analysis results did not survive correction for multiple comparison.

### MOR system

Whole brain voxel-wise analysis revealed higher BP_ND_ in ASD participants compared to controls in the cuneal/precuneal cortex (BA 7,18,19&39, Cluster size 2069 K_E_, cluster defining threshold *p* < 0.05 (FDR-corrected), t = 1.68, Cohen´s d = 0.3) (Figure S1). ROI analysis revealed no overall differences between groups in MOR BP_ND_. However, regional post-hoc analyses indicated 24% lower BP_ND_ for MOR in the hippocampus of ASD group (Estimate 0.214, 100x(e^0.214^ – 1) = 24%, *p* = 0.022, 95% CI [0.039,0.389], Shapiro *p* = 0.381) (Fig. 2, Table S3), but the results did not withstand multiple comparison. BP_ND_ in the hippocampus was also negatively correlated with AQ scores across all participants with one AQ point increase predicting a 1% decrease in NAcc BP_ND_ (Estimate − 0.01, *p* = 0.054, Shapiro *p* = 0.201, 100x(e^−0.01^– 1) = 1%), but this effect was not significant when the groups were analyzed separately (Fig. 3).

### Dopamine-opioid interaction

**Fig. 4:**
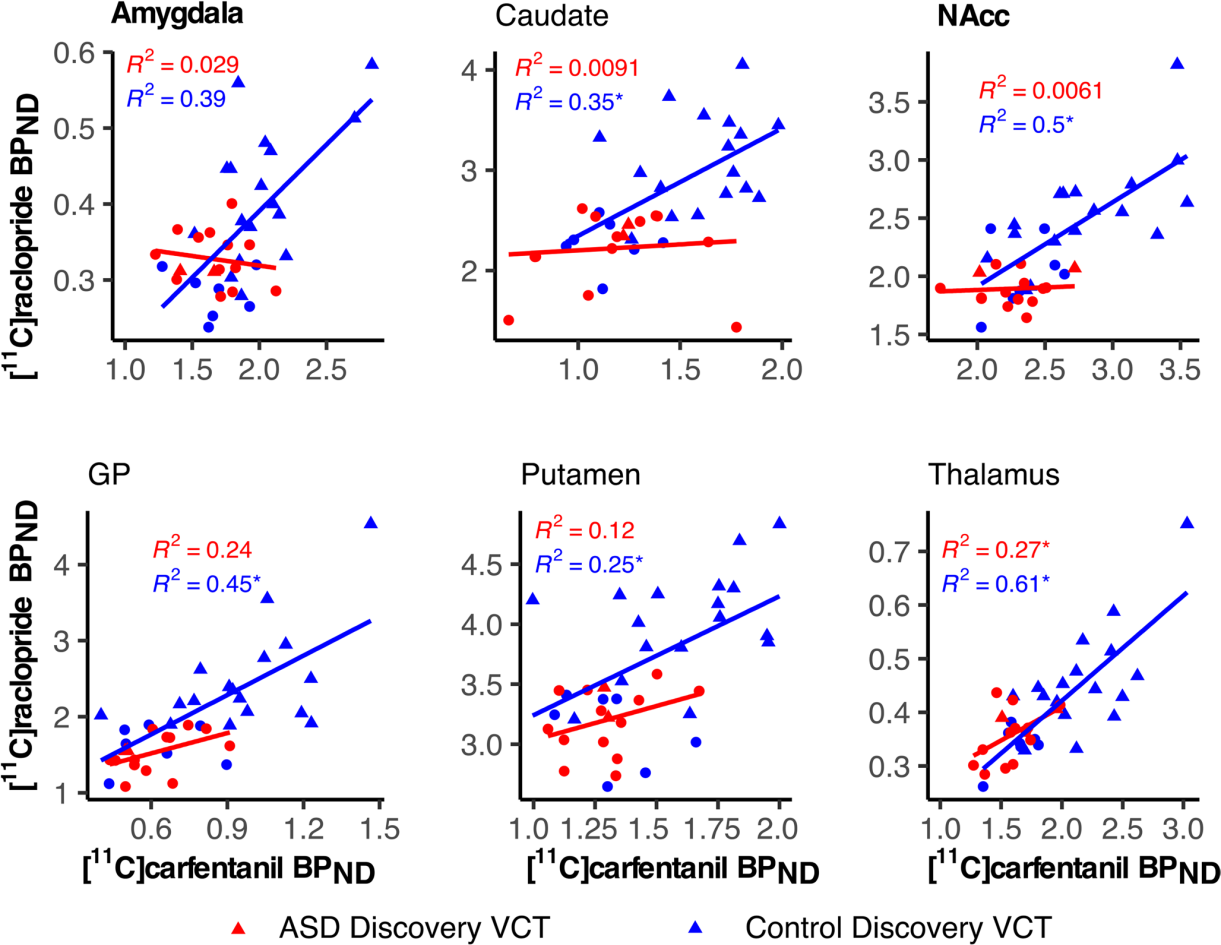
Regional association and LS-regression lines between [11 C]carfentanil and [11 C]raclopride BP_ND_ in control and ASD groups. Significant within-group correlations are marked with an asterisk, and significant differences in correlations between groups (Amygdala and NAcc) with boldface

**Fig. 5:**
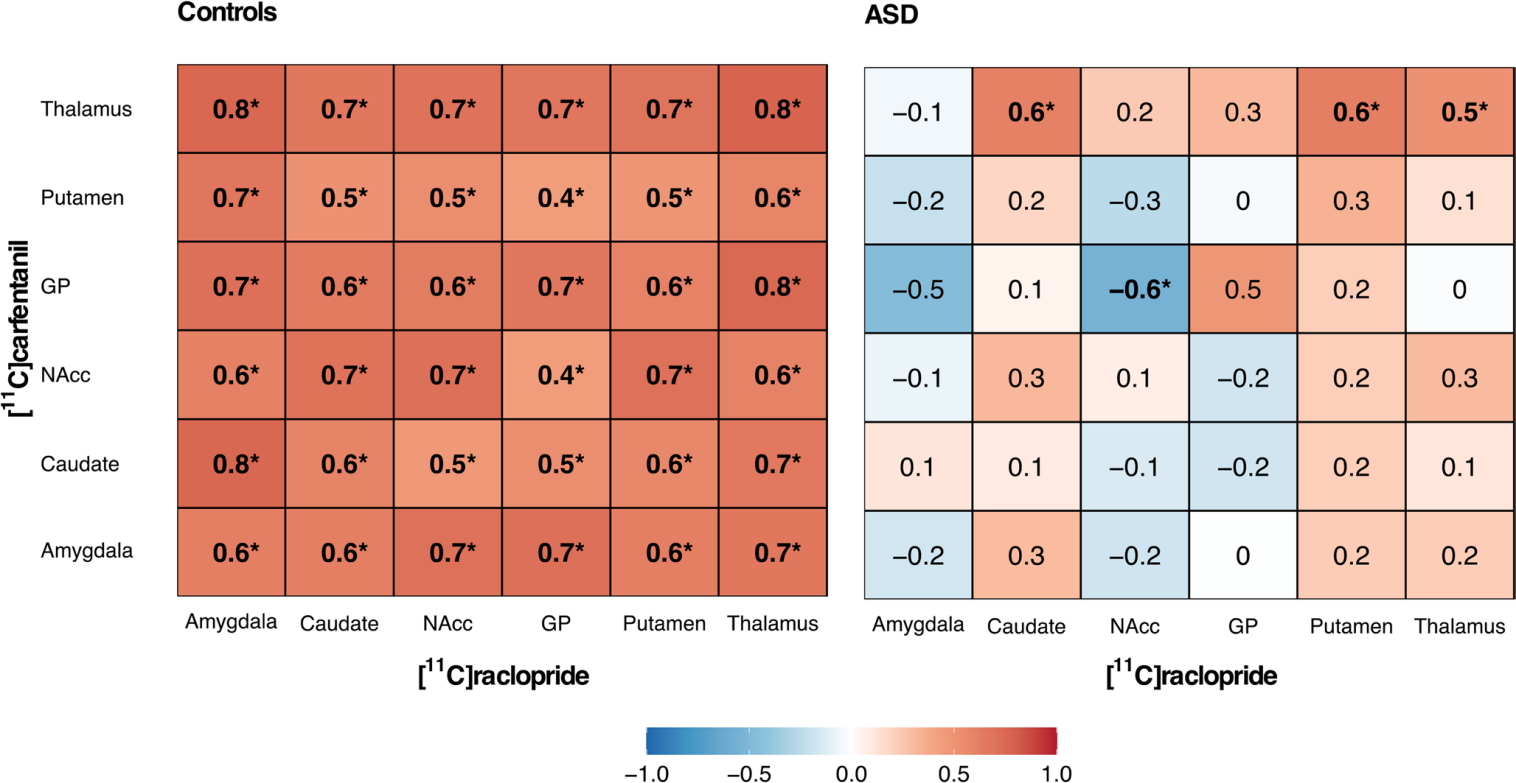
Matrices showing interregional Pearson correlations between [11 C]carfentanil (rows) and [11 C]raclopride (columns) BP_ND_ in the control and ASD groups. Significant correlations are marked with boldface and asterisk

In the control group, the BP_ND_ for [11 C]carfentanil and [11 C]raclopride correlated positively across all six ROIs that were analyzed with both ligands (Figs. 4 and 5). In the ASD group, significant correlation was only observed in thalamus (Figs. 4 and 5). The correlations were significantly stronger in the control versus ASD group in amygdala (z = 2.48 two-tailed *p* = 0.013) and NAcc (z = 2.230, two-tailed *p* = 0.026) (Figs. 4 and 5). The Mantel test did not indicate similarity between the D2R/MOR correlation matrices for control and ASD group (*r* = 0.65, *p* = 0.16).

### Meta-analysis of PET-imaging data on dopamine systems in ASD

**Fig. 6:**
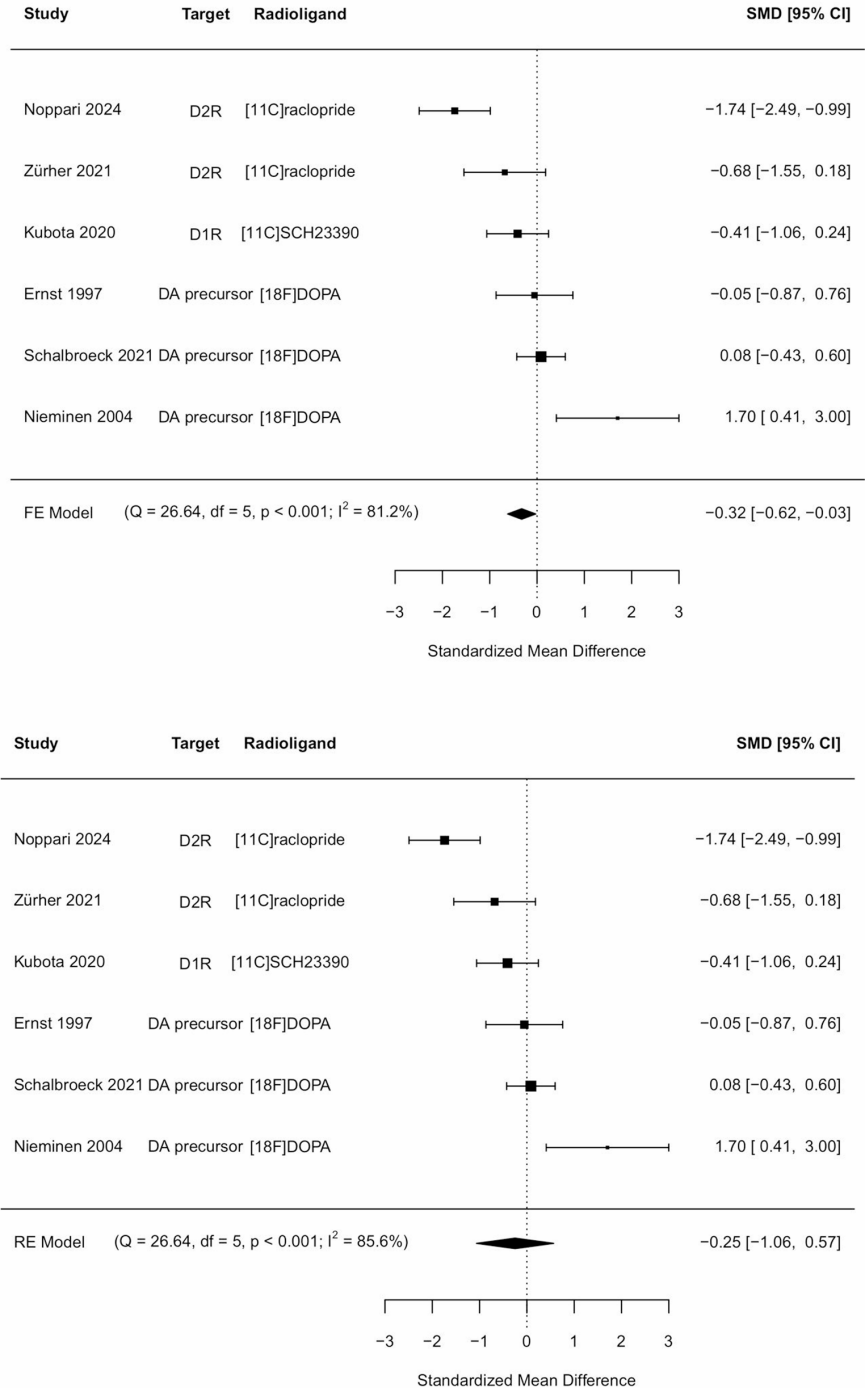
Separate and pooled standardized mean differences (SMDs) in a meta-analysis of six striatal dopamine PET imaging studies with fixed (FE) and random effect (RE) models. Q = Cochran´s Q statistic, df = degrees of freedom, I^2^ = inconsistency index

A fixed effect model showed a standardized mean difference (SMD) of −0.32 (−0.62, −0,03) with heterogeneity of 81.2% (*p* < 0.001), and a random model showed SMD of −0.25 (−1.06, 0.57) with heterogeneity of 85,6% (*p* < 0.001). We found a small overall effect of decreased dopamine tracer binding in ASD compared to controls (Fig. 6).

## Discussion

Our main findings were that ASD is associated with downregulated striatal D2R availability and aberrant interaction between D2R and MOR systems. Alterations in the MOR system were restricted to the hippocampus and left cuneal/precuneal cortex. Altogether these data suggest that the dopaminergic system might be one of the neurotransmitter pathways linked with autism, also partly due to its aberrant interaction with the endogenous MOR system.

### D2R system

Mean BP_ND_ for [11 C]raclopride in the striatal ROIs was lower in the ASD compared to the control group. Our meta-analysis of the dopamine PET studies showed a small and heterogeneous overall effect of dopamine downregulation in ASD compared to controls. The studies using FDOPA reported either no differences or dopamine upregulation in the ASD compared to controls, while the other studies using [11 C]raclopride and [11 C]SCH23390 reported findings similar to ours. Differences in receptor availability, rather than dopamine synthesis capacity (measured by FDOPA), may thus be altered in ASD [93]. Striatal dopamine modulates social and nonsocial reward and motivation [5, 11], which may be disrupted in ASD, according to fMRI findings [21]. In animal studies, the striatal dopaminergic effects have been associated with stereotypic repetitive behavior [94], but the human imaging findings in ASD have not been conclusive [95].

The post-hoc ROI analysis revealed significantly lower BP_ND_ in the NAcc and GP in the ASD group than in the control group, and the AQ scores were also correlated with BP_ND_ in the NAcc across all participants. However, this finding has to be considered preliminary as it did not survive comparison for multiple comparison. In human fMRI studies, NAcc and GP have been associated with responses to reward and punishment [66, 96] and in animal studies, dopamine level in NAcc has been associated with prosocial behavior (increased release) or social avoidance in defeat situations (decreased release) [97].

These present D2R findings could relate, for instance, to aberrant social motivation and potentially explain atypical responses to social reward and high social avoidance in ASD [21, 23, 52, 97]. There are also possible links between striatal function and stereotypic repetitive behavior in ASD [94, 95]. However, none of these findings can be verified within the scope of this study, and further research is needed.

### MOR system

The whole brain voxel-wise analysis showed a significant group difference in the left cuneal/precuneal cortex with higher BP_ND_ in the ASD group. The cuneus plays an essential role in primary visual processing [98]. Atypical connectivity of cuneal/precuneal and temporal areas has been associated to ASD [99, 100]. Precuneus is one of the main areas of the Default Mode Network (DMN) critically involved in social cognition and showing dysfunction in ASD [101–105]. PET studies have shown significant distribution of opioid receptors in the human DMN, and opioids modulate its activity in a dose-dependent manner as the low opioid doses inhibit and the high doses enhance it [28]. Our previous study with a largely overlapping sample revealed lower grey matter volume in the left precuneus of the ASD group [106]. The present study provides novel evidence that besides structural and functional alterations, also MOR functioning is abnormal in the precuneus area in individuals with ASD. This could play a role in explaining the symptom spectrum of ASD.

The post-hoc ROI analyses indicated lower BP_ND_ in the hippocampus in ASD participants compared to controls, and the AQ scores were also correlated with BP_ND_ in the hippocampus across all participants. However, the finding has to be considered preliminary as the group comparison did not withstand multiple comparison. There are fMRI studies demonstrating the involvement of the hippocampus in impaired perception of social emotions, as well as atypical learning, memory, and sensory hypersensitivity in ASD [107]. The hippocampus is also part of the MOR-modulated DMN and has stronger functional connectivity with other DMN regions in ASD compared to controls [107]. Moreover, the animal models of ASD present an impaired hippocampal neurogenesis, which has been found to be modulated by MOR. The more precise regulatory mechanisms are still unclear and the results are controversial [108–110]. Our findings on MOR availability could relate to impairments in social cognition and aberrant modulation of the DMN in ASD.

### D2R and MOR system interaction

The BP_ND_ of [11 C]carfentanil and [11 C]raclopride showed high interligand correlations across the ROIs in the control group, while in the ASD group, significant interligand correlation was found only in the thalamus. Based on the direct comparison of the correlation strengths, the association between the two ligands was significantly stronger in the amygdala and NAcc of the control group compared to ASD group. In animal studies, both neurotransmitters have been associated with the modulation of motivational and rewarding aspects of social interaction [52, 111]. The NAcc has been identified as the key site for this dopamine and opioid modulation of social interaction, and the role of the amygdala has also been recognized in earlier studies [52, 76, 111, 112]. PET studies suggest a largely similar interaction between endogenous opioid and dopamine signaling in humans than observed in animals [47–49]. Interestingly, the interaction of these two neurotransmitters in NAcc is also implicated in the modulation of pain sensations [113, 114] that are both perceived and expressed abnormally in ASD [115]. While the existing knowledge of the aberrant pain modulation in ASD is still scarce [115], the previous and our present findings suggest a possible direction for further investigations.

### Limitations

Despite our modest sample size, we found significant group differences and correlations to ASD symptoms in D2R and MOR systems. A more detailed examination of the role of these two neuromodulatory systems in different ASD symptom domains would require a larger sample size. Scanner hardware and software differences using two different PET scanners may contribute to data variability and could influence the results. However, due to the nested structure of our data, we attempted to statistically control for scanner differences by modelling a random intercept for each scanner. To reduce the heterogeneity in the ASD sample, we included only male participants, and therefore, our results may not be generalizable to the ASD population as a whole [116]. Further research is needed to examine if our findings can be replicated in females. Since ASD is not the only psychiatric disorder with features of social impairment [117], our findings might not be ASD specific and the role of dopaminergic dysfunction across other disorders warrants further studies [118]. Since [11 C]raclopride ligand bind to D2R/D3R and reflects receptor availability reliably only in the striatum, our findings should not be generalized to other mechanisms in the dopamine system (e.g., extra-striatal or D1R dopamine binding). Because D2R and D1R have an opposing role in reward and aversive learning, comparing both of these receptors could be beneficial in the future studies related to dopaminergic reward system and ASD [14, 43]. Since also [11 C]carfentanil binds specifically to MOR, our results should not be generalized to other mechanisms in the opioid system either (DOR, KOR). Moreover, our measurement was restricted to single baseline scans of [11 C]raclopride and [11 C]carfentanil binding, which reflect predominantly tonic receptor availabilities and do not reveal any precise neurotransmitter mechanisms behind them (e.g. postsynaptic/presynaptic, hypo/hyper dopaminergic or opioidergic function). A comparison between basal versus phasic task-related imaging protocols could help obtain more detailed functional interpretation for aberrant D2R and MOR systems in ASD. As our meta-analysis on striatal PET-studies included multiple different dopamine markers with varying functional roles, the results provide only a general overview on the striatal dopaminergic aberrancies in ASD, and additional data is needed for assessing the more specific effects of ASD on dopaminergic pathways.

## Conclusions

Taken together, our findings reveal that ASD is associated with downregulated D2R availability and aberrant dopamine-opioid interaction in the striatum, as well as deviant MOR availability in the areas related to DMN. These findings pave the way for new avenues to study the neuromolecular pathways leading to ASD symptoms and to develop related pharmacological treatments.

## Supplementary information

Below is the link to the electronic supplementary material.

Supplementary File 1(DOCX 30.6 KB)

Supplementary File 2(DOCX 18.5 KB)

Supplementary File 3(DOCX 29.7 KB)

Supplementary File 4(DOCX 199 KB)
