# Supplementary material for: Aberrant type 2 dopamine and mu-opioid receptor availability in autism spectrum disorder
Source: Eur J Nucl Med Mol Imaging. 2025 Oct 18;53(3):2069–82. doi: 10.1007/s00259-025-07620-5 (PMC12860854; doi:10.1007/s00259-025-07620-5)
Supplement: Supplementary file 1 — (DOCX 30.6 KB) [file 259_2025_7620_MOESM1_ESM.docx]

European Journal of Nuclear Medicine and Molecular Imaging

**Aberrant Type 2 Dopamine and mu-Opioid Receptor Availability in Autism Spectrum Disorder**

**Abnormal opioid-dopamine interaction in autism**

MD Tuomo Noppari^1-3^, PhD Jouni Tuisku^1-2^, MD Lasse Lukkarinen^1-2^, Doc Pekka Tani^3^, Prof Nina Lindberg^4^, MSc Emma Saure^5^, Prof Hannu Lauerma^6^, Prof Jari Tiihonen^7-9^, Doc Jussi Hirvonen^10^, MD Semi Helin^11^, Johan Rajander^12^, Prof Juha Salmi^13^, Prof Lauri Nummenmaa^1-2,14^

^1^ Turku PET Centre, University of Turku, Finland, ^2^Turku University Hospital, Turku, Finland, ^3^Department of Psychiatry, Helsinki University Hospital, Finland, ^4^Department of Forensic Psychiatry, Helsinki University Hospital, Finland, ^5^Department of Psychology and Logopedics, Faculty of Medicine, University of Helsinki, Finland, ^6^Psychiatric Hospital for Prisoners, Health Care Services for Prisoners, Turku, Finland, ^7^Department of Clinical Neuroscience, Karolinska Institute and Center for Psychiatry Research, Stockholm, Sweden, ^8^Department of Forensic Psychiatry, University of Eastern Finland, Niuvanniemi Hospital, Kuopio, Finland, ^9^Neuroscience Center, University of Helsinki, Finland, ^10^Department of Radiology, Turku University Hospital, Finland, ^11^Radiopharmaceutical Chemistry Laboratory, Turku PET Centre, University of Turku, Finland, ^12^Turku PET Centre, Acceletor Laboratory, Åbo Akademi University, Turku, Finland, ^13^Unit of Psychology, Faculty of Education and Psychology, University of Oulu, Finland, ^14^Department of Psychology, University of Turku, Finland.

Corresponding author: Tuomo Noppari, Department of Psychiatry, Helsinki University Hospital, PL 590, 00029 HUS, Helsinki, Finland, [tuomo.noppari@hus.fi](mailto:tuomo.noppari@hus.fi), ORCID 0009-0002-1757-082X

**Table S1.** Clinical and sociodemographic characteristics of the participants.

| **Patient** | **Age** | **BMI** | **AQ Score*** | **ADOS Score** | **Diagnosis** | **Medication** | **Education*** | **Handedness** |
| --- | --- | --- | --- | --- | --- | --- | --- | --- |
|  |  |  |  |  |  |  |  |  |
| **ASD** |  |  |  |  |  |  |  |  |
|  |  |  |  |  |  |  |  |  |
| 1 | 29 | 30 | 33 | 16 | ASD | None | Second degree | Left |
| 2 | 31 | 21 | 26 | 7 | ASD | None | University | Left |
| 3 | 27 | 21 | 33 | 10 | ASD | None | Second degree | Right |
| 4 | 26 | 34 | 40 | 7 | ASD, ADHD | None | Second degree | Right |
| 5 | 31 | 30 | 30 | 10 | ASD, MAD | Fluoxetine | Second degree | Right |
| 6 | 27 | 29 | 23 | 7 | ASD, MAD | Zolpidem  (stopped 1 day before) | Second degree | Right |
| 7 | 25 | 30 | 27 | 16 | ASD | None | Second degree | Right |
| 8 | 35 | 41 | 16 | 12 | ASD, MAD | Melatonin | Primary School | Left |
| 9 | 28 | 22 | 32 | 13 | ASD | None | University | Left |
| 10 | 42 | 19 | 23 | 2 | ASD, MAD | Venlafaxine, Mirtazapine | Second degree | Right |
| 11 | 40 | 18 | 34 | 12 | ASD | None | Second degree | Right |
| 12 | 23 | 24 | 21 | 17 | ASD, MAD | Escitalopram | Second degree | Left |
| 13 | 28 | 27 | 24 | 14 | ASD, MAD | None | Second degree | Right |
| 14 | 31 | 19 | 30 | 14 | ASD, ADHD | None | Primary School | Right |
| 15 | 29 | 22 | 27 | 18 | ASD | None | Second degree | Right |
| 16 | 27 | 21 | 29 | 4 | ASD | None | Second degree | Right |
| *Mean (Std.)* | *30*  *(5)* | *26*  *(7)* | *28*  *(6)* | *11*  *(5)* |  |  |  |  |
|  |  |  |  |  |  |  |  |  |
| **Controls** |  |  |  |  |  |  |  |  |
|  |  |  |  |  |  |  |  |  |
| 1 | 30 | 29 | 9 | _- | None | None | Second degree | Right |
| 2 | 21 | 23 | 10 | - | None | None | Second degree | Right |
| 3 | 22 | 25 | 18 | - | None | None | Second degree | Right |
| 4 | 23 | 26 | 15 | - | None | None | Second degree | Right |
| 5 | 21 | 26 | 16 | - | None | None | Second degree | Right |
| 6 | 34 | 23 | 12 | - | None | None | Second degree | Right |
| 7 | 47 | 24 | 12 | - | None | None | University | Left |
| 8 | 23 | 24 | 9 | - | None | None | Second degree | Right |
| 9 | 31 | 25 | 11 | - | None | None | University | Right |
| 10 | 20 | 22 | 14 | - | None | None | Second degree | Right |
| 11 | 43 | 28 | 4 | - | None | None | University | Right |
| 12 | 25 | 27 | 9 | - | None | None | University | Right |
| 13 | 26 | 22 | 6 | - | None | None | University | Left |
| 14 | 28 | 27 | 13 | - | None | None | University | Right |
| 15 | 32 | 22 | 7 | - | None | None | Second degree | Left |
| 16 | 37 | 27 | 11 | - | None | None | University | Right |
| 17 | 33 | 27 | 9 | - | None | None | University | Right |
| 18 | 24 | 25 | 9 | - | None | None | University | Right |
| 19 | 22 | 27 | 14 | - | None | None | Second degree | Right |
| 20 | 22 | 23 | 12 | - | None | None | Second degree | Right |
| 21 | 26 | 24 | 14 | - | None | None | University | Left |
| 22 | 45 | 27 | 18 | - | None | None | Second degree | Right |
| 23 | 49 | 29 | 7 | - | None | None | Second degree | Right |
| 24 | 21 | 26 | 27 | - | None | None | Second degree | Right |
| *Mean (Std.)* | *29*  *(9)* | *25*  *(2)* | *12*  *(5)* |  |  |  |  |  |

Note: ASD=Asperger´s syndrome, ADHD=Attention-Deficit/Hyperactivity disorder, MAD=Mood and Anxiety Disorder.

Statistically significant group differences (p<0.05) are marked with an asterisk.
