# Supplementary material for: Aberrant type 2 dopamine and mu-opioid receptor availability in autism spectrum disorder
Source: Eur J Nucl Med Mol Imaging. 2025 Oct 18;53(3):2069–82. doi: 10.1007/s00259-025-07620-5 (PMC12860854; doi:10.1007/s00259-025-07620-5)
Supplement: Supplementary file 2 — (DOCX 18.5 KB) [file 259_2025_7620_MOESM2_ESM.docx]

European Journal of Nuclear Medicine and Molecular Imaging

**Aberrant Type 2 Dopamine and mu-Opioid Receptor Availability in Autism Spectrum Disorder**

**Abnormal opioid-dopamine interaction in autism**

MD Tuomo Noppari^1-3^, PhD Jouni Tuisku^1-2^, MD Lasse Lukkarinen^1-2^, Doc Pekka Tani^3^, Prof Nina Lindberg^4^, MSc Emma Saure^5^, Prof Hannu Lauerma^6^, Prof Jari Tiihonen^7-9^, Doc Jussi Hirvonen^10^, MD Semi Helin^11^, Johan Rajander^12^, Prof Juha Salmi^13^, Prof Lauri Nummenmaa^1-2,14^

^1^ Turku PET Centre, University of Turku, Finland, ^2^Turku University Hospital, Turku, Finland, ^3^Department of Psychiatry, Helsinki University Hospital, Finland, ^4^Department of Forensic Psychiatry, Helsinki University Hospital, Finland, ^5^Department of Psychology and Logopedics, Faculty of Medicine, University of Helsinki, Finland, ^6^Psychiatric Hospital for Prisoners, Health Care Services for Prisoners, Turku, Finland, ^7^Department of Clinical Neuroscience, Karolinska Institute and Center for Psychiatry Research, Stockholm, Sweden, ^8^Department of Forensic Psychiatry, University of Eastern Finland, Niuvanniemi Hospital, Kuopio, Finland, ^9^Neuroscience Center, University of Helsinki, Finland, ^10^Department of Radiology, Turku University Hospital, Finland, ^11^Radiopharmaceutical Chemistry Laboratory, Turku PET Centre, University of Turku, Finland, ^12^Turku PET Centre, Acceletor Laboratory, Åbo Akademi University, Turku, Finland, ^13^Unit of Psychology, Faculty of Education and Psychology, University of Oulu, Finland, ^14^Department of Psychology, University of Turku, Finland.

Corresponding author: Tuomo Noppari, Department of Psychiatry, Helsinki University Hospital, PL 590, 00029 HUS, Helsinki, Finland, [tuomo.noppari@hus.fi](mailto:tuomo.noppari@hus.fi), ORCID 0009-0002-1757-082X

| **Study** | **Tracer** | **Target** | **ASD**  **n** | **Controls**  **n** | **Age**  **Mean (Std.)** | **Diagnosis** |
| --- | --- | --- | --- | --- | --- | --- |
| *Ernst et al, 1997* | [18F]FDOPA | Precursor | 8 male  6 female | 7 male  3 female | ASD: 13(2) Controls: 14(2) | DSM-III-R |
| *Nieminen von Wendt et al, 2004* | [18F]FDOPA | Precursor | 8 male | 5 male | ASD: 29(6) Controls: 3(5) | DSV-IV,  ICD-10 |
| *Kubota et al, 2020* | [11C]SCH23390 | D1R | 18 male | 20 male | ASD: 33(8) Controls: 30(6) | DSM-IV-TR |
| *Schalbroeck et al, 2021* | [18F]-FDOPA | Precursor | 28 male  16 female | 14 male  8 female | ASD: 24(3), Controls: 23(2) | ADOS-2 AQ |
| *Zürher et al, 2021* | [11C]raclopride | D2R | 10 male | 10 male  2 female | ASD: 25(4) Controls: 26(4) | DSM-V,  ADOS-2 |
| *Noppari et al, 2024* | [11C]raclopride | D2R | 16 male | 24 male | ASD: 30(5)  Controls: 29(9) | DSM-V,  ADOS-2 |

**Table S2.** Imaging methods and participant characteristics for the meta-analysis of striatal dopamine PET studies in ASD.
