# Supplementary material for: Aberrant type 2 dopamine and mu-opioid receptor availability in autism spectrum disorder
Source: Eur J Nucl Med Mol Imaging. 2025 Oct 18;53(3):2069–82. doi: 10.1007/s00259-025-07620-5 (PMC12860854; doi:10.1007/s00259-025-07620-5)
Supplement: Supplementary file 3 — (DOCX 29.7 KB) [file 259_2025_7620_MOESM3_ESM.docx]

European Journal of Nuclear Medicine and Molecular Imaging

**Aberrant Type 2 Dopamine and mu-Opioid Receptor Availability in Autism Spectrum Disorder**

**Abnormal opioid-dopamine interaction in autism**

MD Tuomo Noppari^1-3^, PhD Jouni Tuisku^1-2^, MD Lasse Lukkarinen^1-2^, Doc Pekka Tani^3^, Prof Nina Lindberg^4^, MSc Emma Saure^5^, Prof Hannu Lauerma^6^, Prof Jari Tiihonen^7-9^, Doc Jussi Hirvonen^10^, MD Semi Helin^11^, Johan Rajander^12^, Prof Juha Salmi^13^, Prof Lauri Nummenmaa^1-2,14^

^1^ Turku PET Centre, University of Turku, Finland, ^2^Turku University Hospital, Turku, Finland, ^3^Department of Psychiatry, Helsinki University Hospital, Finland, ^4^Department of Forensic Psychiatry, Helsinki University Hospital, Finland, ^5^Department of Psychology and Logopedics, Faculty of Medicine, University of Helsinki, Finland, ^6^Psychiatric Hospital for Prisoners, Health Care Services for Prisoners, Turku, Finland, ^7^Department of Clinical Neuroscience, Karolinska Institute and Center for Psychiatry Research, Stockholm, Sweden, ^8^Department of Forensic Psychiatry, University of Eastern Finland, Niuvanniemi Hospital, Kuopio, Finland, ^9^Neuroscience Center, University of Helsinki, Finland, ^10^Department of Radiology, Turku University Hospital, Finland, ^11^Radiopharmaceutical Chemistry Laboratory, Turku PET Centre, University of Turku, Finland, ^12^Turku PET Centre, Acceletor Laboratory, Åbo Akademi University, Turku, Finland, ^13^Unit of Psychology, Faculty of Education and Psychology, University of Oulu, Finland, ^14^Department of Psychology, University of Turku, Finland.

Corresponding author: Tuomo Noppari, Department of Psychiatry, Helsinki University Hospital, PL 590, 00029 HUS, Helsinki, Finland, [tuomo.noppari@hus.fi](mailto:tuomo.noppari@hus.fi), ORCID 0009-0002-1757-082X

**Table S3_a_.** Post hoc ROI analysis results. Mean BP_ND_ and standard deviation (SD) in each analyzed region of interest (ROI). Group variances were equal based on Levene's test for both cameras.

| [11C]carfentanil |  |  |  |  |
| --- | --- | --- | --- | --- |
|  |  |  |  |  |
| *Discovery D690* |  |  |  |  |
|  | **Controls** | | **ASD** | |
| ROI | **Mean BP_ND_** | **SD** | **Mean BP_ND_** | **SD** |
| globus pallidus (GP) | 0.625 | 0.170 | 0.617 | 0.140 |
| amygdala | 1.669 | 0.239 | 1.653 | 0.255 |
| caudatus | 1.141 | 0.164 | 1.145 | 0.332 |
| dorsal anterior cingulate cortices (dACC) | 1.154 | 0.116 | 1.051 | 0.256 |
| insula | 1.051 | 0.096 | 1.042 | 0.125 |
| nucleus accumbens (NAcc) | 2.346 | 0.236 | 2.230 | 0.209 |
| orbitofrontal cortex | 0.970 | 0.084 | 0.947 | 0.138 |
| posterior cingulate cortices (PCC) | 0.873 | 0.082 | 0.841 | 0.195 |
| putamen | 1.322 | 0.194 | 1.277 | 0.180 |
| rostal anterior cingulate cortices (rACC) | 1.180 | 0.155 | 1.160 | 0.198 |
| thalamus | 1.629 | 0.153 | 1.577 | 0.183 |
| hippocampus | 0.377 | 0.073 | 0.367 | 0.075 |
|  |  |  |  |  |
| *Discovery VCT* |  |  |  |  |
|  | **Control** | | **ASD** | |
| ROI | **Mean BP_ND_** | **SD** | **Mean BP_ND_** | **SD** |
| globus pallidus (GP) | 0.963 | 0.254 | 0.502 | 0.006 |
| amygdala | 2.022 | 0.331 | 1.536 | 0.176 |
| caudatus | 1.614 | 0.245 | 1.234 | 0.016 |
| dorsal anterior cingulate cortices (dACC) | 1.337 | 0.261 | 1.185 | 0.011 |
| insula | 1.247 | 0.240 | 1.074 | 0.056 |
| nucleus accumbens (NAcc) | 2.796 | 0.468 | 2.369 | 0.497 |
| orbitofrontal cortex (OFC) | 1.174 | 0.237 | 1.122 | 0.045 |
| posterior cingulate cortices (PCC) | 1.057 | 0.219 | 0.996 | 0.104 |
| putamen | 1.606 | 0.287 | 1.296 | 0.012 |
| rostal anterior cingulate cortices (rACC) | 1.426 | 0.314 | 1.346 | 0.147 |
| thalamus | 2.178 | 0.365 | 1.731 | 0.316 |
| hippocampus | 0.537 | 0.121 | 0.288 | 0.033 |
|  |  |  |  |  |
|  |  |  |  |  |
|  |  |  |  |  |
| [^11^C]raclopride |  |  |  |  |
|  |  |  |  |  |
| *Discovery D690* |  |  |  |  |
|  | **Controls** | | **ASD** | |
| ROI | **Mean BP_ND_** | **SD** | **Mean BP_ND_** | **SD** |
| caudatus | 2.272 | 0.239 | 2.231 | 0.406 |
| nucleus accumbens (NAcc) | 2.025 | 0.312 | 1.895 | 0.148 |
| globus pallidus (GP) | 1.609 | 0.291 | 1.476 | 0.228 |
| putamen | 3.119 | 0.314 | 3.221 | 0.283 |
| amygdala | 0.283 | 0.032 | 0.330 | 0.036 |
| thalamus | 0.339 | 0.038 | 0.367 | 0.049 |
|  |  |  |  |  |
| *Discovery VCT* |  |  |  |  |
|  | **Controls** | | **ASD** | |
| ROI | **Mean BP_ND_** | **SD** | **Mean BP_ND_** | **SD** |
| caudatus | 3.098 | 0.473 | 2.180 | 0.057 |
| nucleus accumbens (NAcc) | 2.547 | 0.443 | 1.880 | 0.025 |
| globus pallidus (GP) | 2.478 | 0.685 | 1.864 | 0.038 |
| putamen | 4.025 | 0.438 | 3.072 | 0.076 |
| amygdala | 0.415 | 0.087 | 0.309 | 0.035 |
| thalamus | 0.461 | 0.099 | 0.317 | 0.019 |

**Table S3_b_.** Results of the LMM analysis for log-transformed BP_ND_ values. Group Estimates, 95% Confidence Intervals, p-values and Shapiro p for each analyzed region of interest (ROI).

| [11C]carfentanil |  |  |  |  |  |
| --- | --- | --- | --- | --- | --- |
|  |  |  |  |  |  |
| ROI | **Estimate (group)** | **95% CI lower** | **95% CI higher** | **p-value** | **Shapiro p** |
| globus pallidus (GP) | 0.2 | -0.011 | 0.412 | 0.073 | 0.729 |
| amygdala | 0.1 | -0.016 | 0.217 | 0.102 | 0.618 |
| caudatus | 0.106 | -0.058 | 0.27 | 0.216 | 0.914 |
| dorsal anterior cingulate cortices (dACC) | 0.164 | -0.013 | 0.341 | 0.085 | 0 |
| insula | 0.062 | -0.05 | 0.174 | 0.29 | 0.749 |
| *nucleus accumbens (NAcc)* | 0.094 | -0.009 | 0.196 | 0.082 | 0.784 |
| orbitofrontal cortex (OFC) | 0.044 | -0.08 | 0.169 | 0.492 | 0.479 |
| posterior cingulate cortices (PCC) | 0.085 | -0.082 | 0.253 | 0.327 | 0.001 |
| putamen | 0.096 | -0.026 | 0.218 | 0.133 | 0.725 |
| rostal anterior cingulate cortices (rACC) | 0.046 | -0.092 | 0.183 | 0.523 | 0.639 |
| thalamus | 0.095 | -0.013 | 0.203 | 0.096 | 0.904 |
| hippocampus | 0.214 | 0.039 | 0.389 | 0.022 | 0.381 |
|  |  |  |  |  |  |
| [^11^C]raclopride |  |  |  |  |  |
|  |  |  |  |  |  |
| ROI | **Estimate (group)** | **95% CI lower** | **95% CI higher** | **p-value** | **Shapiro p** |
| caudatus | 0.111 | -0.016 | 0.238 | 0.096 | 0.459 |
| nucleus accumbens (NAcc) | 0.118 | 0.013 | 0.222 | 0.034 | 0.062 |
| globus pallidus | 0.175 | 0.008 | 0.343 | 0.048 | 0.23 |
| putamen | 0.041 | -0.041 | 0.123 | 0.335 | 0.32 |
| amygdala | -0.025 | -0.163 | 0.113 | 0.723 | 0.909 |
| thalamus | 0.016 | -0.111 | 0.142 | 0.811 | 0.085 |
|  |  |  |  |  |  |
