# Supplementary material for: Aberrant type 2 dopamine and mu-opioid receptor availability in autism spectrum disorder
Source: Eur J Nucl Med Mol Imaging. 2025 Oct 18;53(3):2069–82. doi: 10.1007/s00259-025-07620-5 (PMC12860854; doi:10.1007/s00259-025-07620-5)
Supplement: Supplementary file 4 — (DOCX 199 KB) [file 259_2025_7620_MOESM4_ESM.docx]

European Journal of Nuclear Medicine and Molecular Imaging

**Aberrant Type 2 Dopamine and mu-Opioid Receptor Availability in Autism Spectrum Disorder**

**Abnormal opioid-dopamine interaction in autism**

MD Tuomo Noppari^1-3^, PhD Jouni Tuisku^1-2^, MD Lasse Lukkarinen^1-2^, Doc Pekka Tani^3^, Prof Nina Lindberg^4^, MSc Emma Saure^5^, Prof Hannu Lauerma^6^, Prof Jari Tiihonen^7-9^, Doc Jussi Hirvonen^10^, MD Semi Helin^11^, Johan Rajander^12^, Prof Juha Salmi^13^, Prof Lauri Nummenmaa^1-2,14^

^1^ Turku PET Centre, University of Turku, Finland, ^2^Turku University Hospital, Turku, Finland, ^3^Department of Psychiatry, Helsinki University Hospital, Finland, ^4^Department of Forensic Psychiatry, Helsinki University Hospital, Finland, ^5^Department of Psychology and Logopedics, Faculty of Medicine, University of Helsinki, Finland, ^6^Psychiatric Hospital for Prisoners, Health Care Services for Prisoners, Turku, Finland, ^7^Department of Clinical Neuroscience, Karolinska Institute and Center for Psychiatry Research, Stockholm, Sweden, ^8^Department of Forensic Psychiatry, University of Eastern Finland, Niuvanniemi Hospital, Kuopio, Finland, ^9^Neuroscience Center, University of Helsinki, Finland, ^10^Department of Radiology, Turku University Hospital, Finland, ^11^Radiopharmaceutical Chemistry Laboratory, Turku PET Centre, University of Turku, Finland, ^12^Turku PET Centre, Acceletor Laboratory, Åbo Akademi University, Turku, Finland, ^13^Unit of Psychology, Faculty of Education and Psychology, University of Oulu, Finland, ^14^Department of Psychology, University of Turku, Finland.

Corresponding author: Tuomo Noppari, Department of Psychiatry, Helsinki University Hospital, PL 590, 00029 HUS, Helsinki, Finland, [tuomo.noppari@hus.fi](mailto:tuomo.noppari@hus.fi), ORCID 0009-0002-1757-082X

**Supplementary figure S1.**


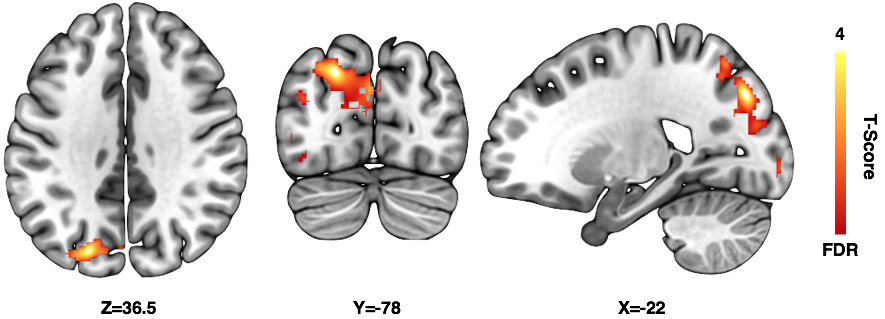


Figure S1. Higher [11C]carfentanil BP_ND_ in the left cuneus/precuneus of ASD participants compared to controls in the whole brain voxel-wise analysis. BA 7,18,19,39, Cluster size 2069 K_E_, cluster defining threshold p< 0.05 (FDR-corrected), t = 1.68, Cohen´s d = 0.3.
